# Prevalence of Hepatitis B Virus Infection in Shenzhen, China, 2015–2018

**DOI:** 10.1038/s41598-019-50173-5

**Published:** 2019-09-26

**Authors:** Jian Tao, Weimin Zhang, Huakui Yue, Guohun Zhu, Wenyuan Wu, Wenbo Gong, Honghui Fang, Guirong He, Xiaoyun Hu, Hongyue Zhao, Aiqin Liu

**Affiliations:** 1 Department of Laboratory Medicine, Shenzhen People’s Hospital, Second Clinical Medical College of Ji’nan University, 1017 Dongmenbei Road, Luohu District, Shenzhen, Guangdong 518020 China; 2 School of Information Technology and Electrical Engineering, University of Queensland, Brisbane, QLD 4067 Australia

**Keywords:** Hepatitis B, Epidemiology

## Abstract

China has nearly 10% of the general HBV carrier population in the world; this infection is the most common cause of chronic liver disease. Understanding HBV epidemiology is essential for future infection control, evaluation, and treatment. This study determined the prevalence of HBV infection in Shenzhen by serological testing and analysis in 282,166 HBV screening cases for the following: HBcAb, indicative of previous HBV infection; HBsAg, indicative of chronic (current) infection; HBsAb, indicative of immunity from vaccination; and 34,368 HBV etiological screening cases for HBV-DNA, indicative of virus carriage, in which 1,204 cases were genotyped and mutation analyzed for drug-resistance evaluation. Shenzhen was a highly endemic area of HBV throughout the study period (prevalence 9.69%). HBV infections were almost entirely in the 20 and older age groups with a male-to-female ratio of 1.16:1 which is approximately the same as the male-to-female ratio of the general population in China. However, only 71.25% of the general population retained HBV immune protection. Genotype B and C were identified as the most common agents; recombinant B/C and B/D also existed; some cases, however, could not be genotyped. NAs resistant mutation occurrence patterns were multitudinous; single mutation patterns of rtM204I/V and rtL180M occurrences accounted for majority, followed by the combinational mutation pattern L180M + M204I/V. Drug-resistance was prevalent, mainly occurring in the cross resistance patterns LAM + LdT and LAM + LdT + ETV, and significantly more critical in males. These results demonstrate that all people free from HBV infection should obtain injections of the vaccine or booster shots, and conventional virologic detection in a clinical laboratory center should incorporate genotype and mutation alongside the serological factors for etiology and develop better classification methods, such as sequencing.

## Introduction

Hepatitis B virus (HBV) infection remains one of the most urgent major public health priorities; it is a devastating cause of morbidity and mortality, accounting for more than 300,000 deaths per year in China^1,2^, despite the fact that the expansion of national immunization programs and the initiation of universal Hepatitis B virus (HBV) vaccination in all newborns has resulted in a steady decline in the prevalence of HBV since 2002^3^. It is currently estimated that 30 million people are chronic carriers of the virus, which is transmitted to others through high-risk behaviors involving percutaneous or mucosal contact with infected blood or other body fluids^1^. If left unmonitored or untreated, approximately one-third of these chronically infected individuals will develop terminal liver disease, such as cirrhosis or HBV-related HCC (HBV-HCC)^4–7^.

Currently-available treatments for HBV have demonstrated their ability to delay disease progression and reduce the incidence of HCC with interferon and the nuceleos(t)ide analogs (NAs) lamivudine (LAM), adefovir (ADV), entecavir (ETV), emtricitabine (TDF), telbivudine (LdT), tenofovir disoproxil fumarate (TDF), and recently FDA-approved tenofovir alafenamide (TAF)^8^. Many patients, however, cannot achieve the ultimate treatment goal of the loss of HBsAg and/or seroconversion to HBsAb and undetectable HBV DNA in the serum^9^, all of which are considered to be indicators of successful therapy (functional cure). Unsuccessful treatment is mostly attributable to drug-resistance after long-term use of NAs^10^.

Sequential NA monotherapy can select for multidrug-resistant HBV strains with mutations in the reverse transcriptase (RT) region of the polymerase gene, which can modify the amino acid sequence of the hepatitis B surface antigen (HBsAg)^11^. These HBV strains, if carrying mutations, would definitely result in treatment failure^12^. Moreover, some of the mutations in HBsAg, such as D144A, Q129R, and G145R, could reduce binding to HBsAbs from the hepatitis B vaccination, bypassing the neutralizing activity of these antibodies (antibody escape) and result in infecting HBV-vaccinated individuals^13^. A total of 8 HBV classical mutation sites are conventionally tested for HBV patients in most clinical labs, including M204I/V, L180M, T184A/F/I/L/S, L181T/V, M250I/L/V, M236T, S202G, and V207I. Early detection of mutants and frequent monitoring of viral loads is crucial for clinicians to select NAs for HBV patients in order to achieve successful therapy outcomes.

HBV genotype characteristics are also essential for disease evaluation and treatment^14^. There are at least 10 HBV genotypes (A to J), and their subtypes have been defined. Different genotypes distribute differently worldwide, whereas in China, there is a prevalence of genotypes B, C, and D^15,16^. Many studies have concluded that the prevalence of HBV genotypes exhibit different clinical features, which suggests it was a determinant of the outcome after acute HBV infection and of chronic HBV infection^17,18^. HBV genotypes were also demonstrated to correlate with the diversity of liver disease and the oxidative damage degree in patients with HBV-induced liver disease^19,20^.

Current clinical HBV diagnostic tests usually only include HBsAg, antibody to hepatitis B surface antigen (HBsAb), hepatitis B e antigen (HBeAg), antibody to hepatitis B e antigen (HBeAb) and antibody to hepatitis B core antigen (HBcAb) quantification; while some patients may refered for HBV-DNA quantification, these seldom involve genotype classification or drug-resistance mutation detection. HBV epidemiology and virologic characteristics (genotypes and mutations) based on a large sample size from Shenzhen has rarely been investigated.

In this study, we have included a total of 282,166 cases from Aug. 26, 2015 to Sept. 11, 2018, of patients who received HBV screening tests to determine HBV epidemiology and etiology, including gender, age, virus genotype, and especially drug-resistance mutations. We estimated the positive rate of age-specific cases of HBV by applying distribution in positive samples in the corresponding serological factors group, respectively. To quantify genotype patterns of HBV by age and gender, we applied age-specific and gender-specific case distribution in each genotype of the HBV. To quantify every single mutation site, we applied age-specific and gender-specific case distributions in each mutation group. To quantify HBV drug-resistant mutation occurrence patterns, we applied those case distributions in each HBV genotype group. To quantify patterns of NAs resistance in HBV patients, we applied those case distributions in each HBV genotype group. These investigations will help to assess the burden of the diseases and the effects of ongoing prevention campaigns, and will be indispensable for the development of future strategic plans.

## Materials and Methods

### Laboratory HBV related tests

Serum samples were collected and tested for HBsAg, HBsAb, HBeAg, HBeAb, and HBcAb by the method of enzyme linked immunosorbent assay (ElASA), using the commercial kit by Kehua Bio-engineering, China; HBV-DNAs by One-step RT-PCRs, using the HBV test commercial kit by Sansure Biotech, China; HBV genotypes and mutations by dot blot hybridization, using a commercial kit by Yaneng Biosciences, China.

### Clinical and laboratory data collection

Shenzhen People’s Hospital, Second Clinical Medical College of Ji’nan University, is the first established comprehensive hospital in Shenzhen. Between Aug. 26, 2015 and Sept. 11, 2018, 282,166 patients received laboratory HBV screening detection and 27,336 patients were identified as HBsAg positive. The clinical and laboratory data was extracted from medical records, including basic demographic information (sex and date of birth), date of illness onset, sample type (serum), symptoms, date of symptoms onset, diagnosis (if applicable), date of diagnosis, date of sampling, method used for HBV test, results at the time of sampling (positive or negative), HBV-DNA level, virus genotype (wild type, B, C, D, mixed, or unclassified), and mutation (no mutation, rt204I, rt180M, rt204V, rt236T, rt250V, rt181V, rt202G, rt250L, rt181T, rt184I, rt184L, rt207I, rt184S, rt250I, rt184A, or rt184F).

Ethical approval for this study was obtained from the Institutional Review Board of Shenzhen People’s Hospital, Second Clinical Medical College of Ji’nan University. All information and patient identifiers were kept anonymous to protect patient confidentiality. Since all data obtained was de-identified, and no extra samples were required, written consent from the patients was waived.

### Data analysis

We included all 282,166 cases from Aug. 26, 2015 to Sept. 11, 2018 in the analysis. We estimated the number of previous (past and present) HBV infection cases by HBcAb, the number of chronic (current) HBV infection by HBsAg, the number of vaccine-induced protection by HBsAb, and the number of HBV carriers by HBV-DNA, respectively. We also evaluated the positive rate of age-specific cases of HBV by applying distribution to the positive samples in the corresponding serological factors group, respectively. To quantify genotype patterns of HBV by age and gender, we applied age-specific and gender-specific case distributions in each genotype of the HBV. To quantify each mutation site, we applied age-specific and gender-specific case distributions in each mutation group. To quantify HBV drug-resistant mutation occurrence patterns, we applied those case distributions in each HBV genotype group. To quantify patterns of NAs resistance in HBV patients, we applied those case distributions in each HBV genotype group.

We used SPSS (version 19.0) to do the analysis.

## Results

**Figure 1:**
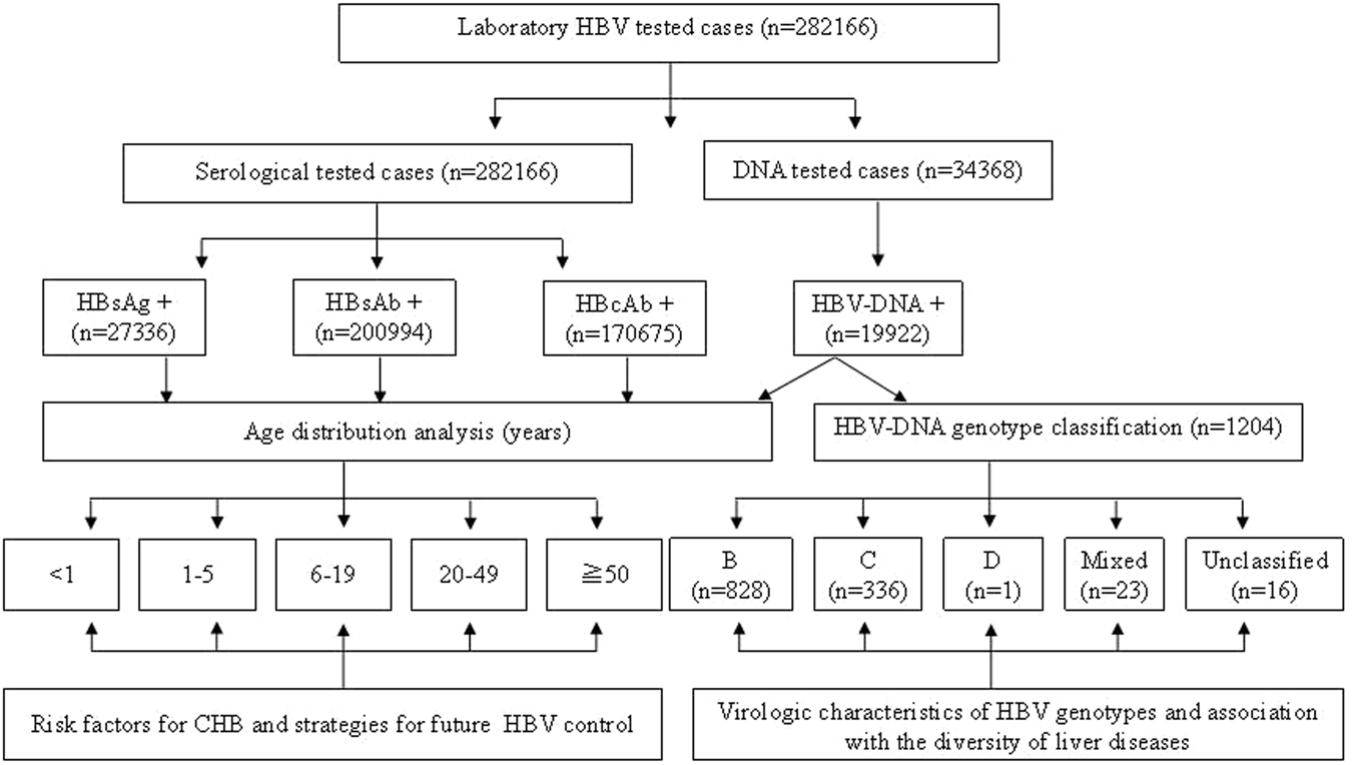
Flow Diagram of HBV patients in whom epidemiology was investigated.

A total of 282,166 people who were admitted to Shenzhen People’s Hospital, Second Clinical Medical College of Ji’nan University, between Aug. 26, 2015 and Sept. 11, 2018, received HBV serological screening tests, of which 27,336 (9.69%) were HBsAg positive cases, 170,675 (60.49%) were HBcAb positive cases, and 200,994 (71.23%) were HBsAb positive cases. A total of 34,368 cases received HBV-DNA tests using serum, of which 19,922 (57.97%) tested positive (Fig. 1).

**Figure 2:**
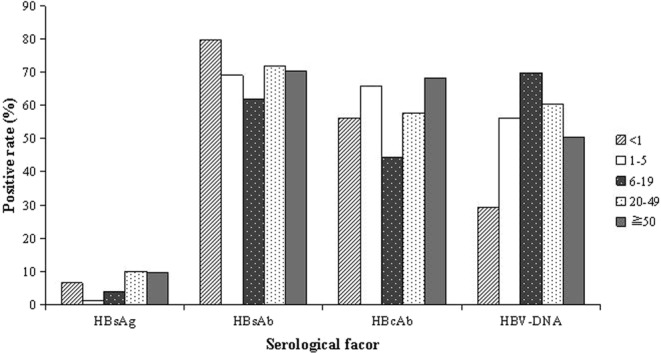
HBV serological factors detection by age group in residents of Shenzhen, China, 2015–18.

The incidence of HBV infection had a male-to-female ratio of 1.16:1 of all HBsAg positive cases throughout the three-year period included in the study which is approximately the same as the male-to-female ratio of the general population in China. The overall prevalence of HBV infection had remained high, up to 9.69%. These infections were almost entirely in the 20–49 age group and 50+ age group, accounting for 73.19% (20007/27336) and 25.47% (6960/27336), respectively. The incidence was very low the 6–19 age group, accounting for only 1.13% (310/27336); and as expected, HBV infection among those of ages less than 6 years was rare, with only 59 positive cases (Table 1). Among patients more than 20 years old, the positive rate of HBsAg reached 9.89% (26967/272557); it was 1.10% (9/815) in the 1–5 age group, 3.87% (310/8014) in the 6–19 age group, and 6.42% (50/779) in the younger than 1 age group (Fig. 2). The data for age groups under 20 years could just reflect the circumstances at Shenzhen People’s Hospital, where sample sizes for children are considerably smaller than the adult groups, presumably because only a few children or teenage patients came to this adult hospital instead of a children’s hospital.

**Table 1:** Prevalence of HBV serological factors by selected characteristics in Shenzhen, China, 2015–18.

Characteristic	HBsAg		HBsAb		HBcAb		HBV-DNA	
	Sample Size	No. Positive Tests	Sample Size	No. Positive Tests	Sample Size	No. Positive Tests	Sample Size	No. Positive Tests
**Overall**	282165	27336	282116	200994	282166	170675	34368	19922
**Sex**								
Male	131120	14661	131101	94187	131062	80582	19899	11661
Female	151045	12675	151015	106807	151104	90093	14469	8261
**Age, years**								
<1	779	50	779	621	779	438	185	54
1–5	815	9	815	563	815	536	32	18
6–19	8014	310	8014	4967	8014	3552	444	310
20–49	201079	20007	201036	144625	201080	116274	25679	15503
≧50	71478	6960	71472	50218	71478	48893	8028	4037

The overall prevalence of past/present HBV remained high up to 60.49% (170675/282166) of all HBcAb positive cases included in the study in this three-year period. It was slightly lower in males than in females with a male-to-female ratio of 0.89:1 of all HBcAb positive cases included in the study. All age groups had high HBV infection history rates, 56.23% in the less than 1 age group, 65.77% in the 1–5 age group, 44.32% in the 6–19 age group, 57.82% in the 20–49 age group, and 68.40% in the 50+ age group (Table 1).

In summary, 71.25% of patients retained HBV immune protection, and there was no significant difference between genders. The less than 1 age group had the highest immune protection rate of 79.72% as a result of implementing vaccinations, while the 1–19 age group showed a decrease to 61.98%, which could be attributed to the natural decline in HBsAb. Adults more than 20 years of age had slightly increased immune protection, which was probably because of previous infection (Table 1).

**Figure 3:**
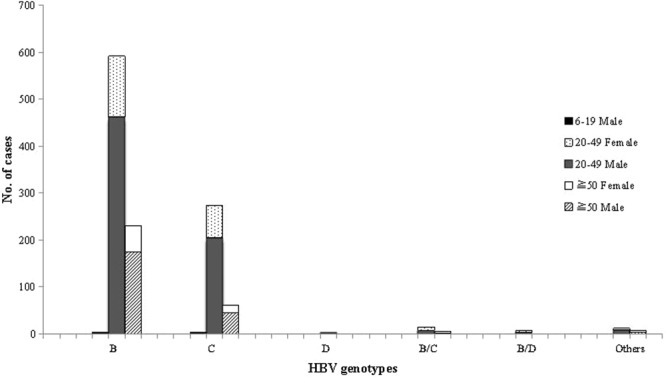
HBV genotypes distribution by age group in residents of Shenzhen, China, 2015–18.

A total of 1204 patients who tested serum HBV DNA positive received an HBV genotype classification. Among them, 828 (68.77%) patients were infected with genotype B, 336 (27.90%) with genotype C, 1 (0.08%) with genotype D, 18 (1.50%) with recombinant B/C, 5 (0.42%) with recombinant B/D, and the remaining 16 (1.33%) with an unclassified genotype (Table 2). In the subgroup analysis, stratified by age and gender, similar patterns of HBV genotype distribution were observed (Fig. 3). Males were significantly higher than female in all genotypes, accounting for 76.16% (917/1204).

**Table 2:** Prevalence of HBV genotypes and mutations in Shenzhen, China, 2015–18.

Age, years	<1		1–5		6–19		20–49		≧50	
Sex	Male	Female	Male	Female	Male	Female	Male	Female	Male	Female
**Genotypes**										
B	0	0	0	0	4	2	462	130	174	56
C	0	0	0	0	1	0	205	68	45	17
D	0	0	0	0	0	0	1	0	0	0
mixed B + C	0	0	0	0	0	0	8	7	2	1
mixed B + D	0	0	0	0	0	0	3	2	0	0
Unclassified	0	0	0	0	0	0	9	3	3	1
**Mutations**										
rt204I	0	0	0	0	0	0	67	14	40	12
rt180M	0	0	0	0	1	0	39	8	15	4
rt204V	0	0	0	0	1	0	23	7	11	3
rt236T	0	0	0	0	0	0	13	2	9	2
rt250V	0	0	0	0	0	0	11	4	2	0
rt181V	0	0	0	0	0	0	9	1	2	3
rt202G	0	0	0	0	0	0	7	4	3	0
rt250L	0	0	0	0	0	0	7	1	5	0
rt181T	0	0	0	0	0	0	6	0	1	0
rt184I	0	0	0	0	0	0	1	0	2	0
rt184L	0	0	0	0	0	0	3	0	0	0
rt207I	0	0	0	0	0	0	1	0	0	1
rt184S	0	0	0	0	0	0	0	1	0	0
rt250I	0	0	0	0	0	0	1	0	0	0
rt184A	0	0	0	0	0	0	0	0	0	0
rt184F	0	0	0	0	0	0	0	0	0	0

**Figure 4:**
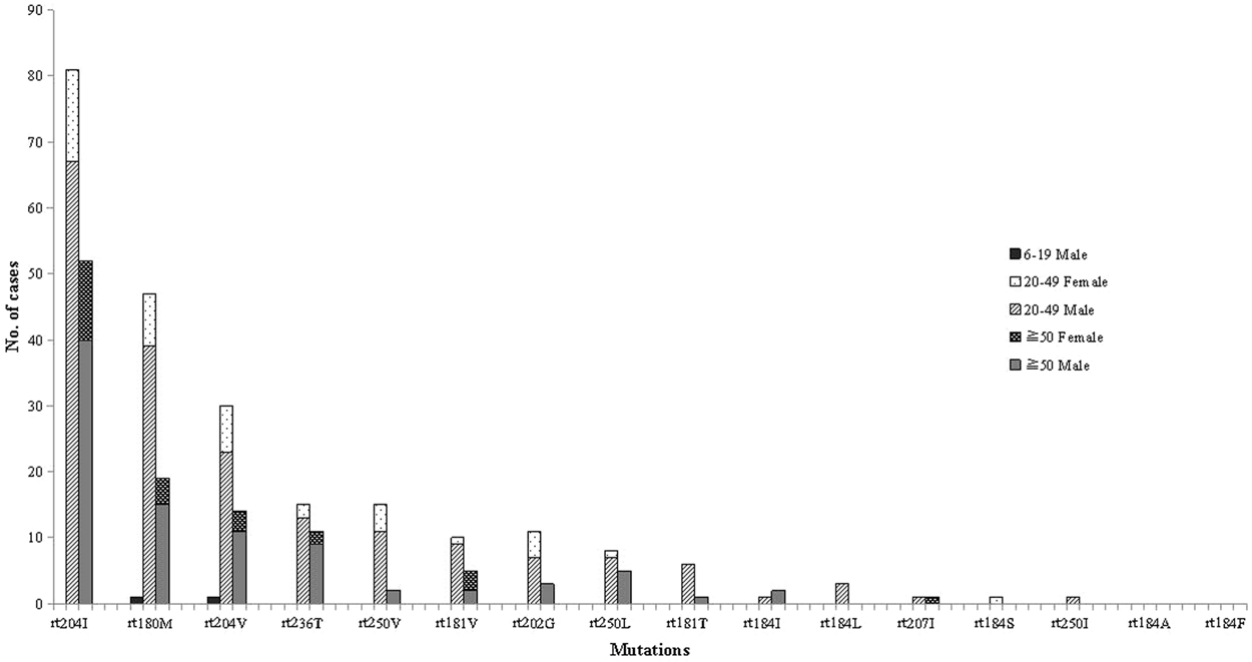
HBV mutations distribution by age group in residents of Shenzhen, China, 2015–18.

In addition to genotyping, all 1204 patients received HBV antiviral resistant mutations detection. A total of 347 mutation occurrences were identified (Table 2). The stratified analysis showed that these mutations mostly occurred in male patients and almost entirely in the 20–49 and 50+ age groups, accounting for 80.70% (280/347) and 99.42% (345/347), respectively. Among them, mutation rtM204I associated with LAM and LdT had the highest proportion, 38.33%; mutation rtL180M associated with LAM, LdT, and ETV accounted for 19.30%; mutation rtM204V associated with LAM, LdT, and ETV accounted for 12.97%; and other mutations accounted for no more than 10% (Fig. 4).

In some cases, mutations were present in combinational patterns, so those 347 mutations occurred in only 205 cases. Total of 38 NAs resistant mutation occurrence patterns were present in this study (Table 3), including 10 single mutation patterns and 28 combinational mutation patterns. Cases with single site mutation accounted for 61.46% (126/205) and cases with combinational mutations accounted for 38.54% (79/205). Single site mutation occurrences were mainly M204I (86/126) and M236T (14/126). Combinational mutation occurrences were mainly L180M + M204I/V (33/79). Other occurrences of single site or combinational mutation patterns were very few and no more than 10, respectively.

**Table 3:** Distribution of HBV drug-resistant mutation occurrence patterns in different genotypes in Shenzhen, China, 2015–18.

Genotypes/Mutation occurrence patterns	B	C	D	mixed B + C	mixed B + D	Unclassified
rt204I	66	21	0	1	1	0
rt236T	12	2	0	0	0	0
rt180M + rt204V	11	3	0	0	0	0
rt180M + rt204I	6	12	0	0	0	1
rt181V + rt236T	6	1	0	0	0	1
rt180M + rt204V + rt202G	4	2	0	0	0	0
rt204V	4	2	0	0	0	0
rt180M + rt204V + rt204I	3	0	0	0	0	0
rt181V	3	1	0	0	0	0
rt204I + rt250L	3	0	0	0	0	0
rt250V	3	1	0	0	0	0
rt204I + rt204V	2	0	0	0	0	0
rt180M + rt204V + rt204I + rt250V + rt236T + rt181V	1	0	0	0	0	0
rt180M + rt204V + rt202G + rt184L	1	0	0	0	0	0
rt180M + rt204V + rt184L	1	0	0	0	0	0
rt180M + rt204V + rt204I + rt250L	1	0	0	0	0	0
rt180M + rt204I + rt250V	1	0	0	0	0	0
rt204V + rt181V + rt202G	1	0	0	0	0	0
rt204I + rt184I	2	1	0	0	0	0
rt204V + rt184L	1	0	0	0	0	0
rt202G	1	1	0	0	0	0
rt204V + rt202G	1	0	0	0	0	0
rt202G + rt204V + rt250V	1	0	0	0	0	0
rt204I + rt236T	1	0	0	0	0	0
rt204I + rt250V	1	0	0	0	0	0
rt250L	1	1	0	0	0	0
rt180M	0	5	0	0	0	0
rt180M + rt204I + rt181T + rt236T	0	1	0	0	0	0
rt180M + rt204V + rt184S	0	1	0	0	0	0
rt180M + rt204I + rt204V + rt202G	0	1	0	0	0	0
rt180M + rt204V + rt250V	0	1	0	0	0	0
rt181T	0	1	0	0	0	0
rt181T + rt204I	0	1	0	0	0	0
rt181T + rt250I + rt250L + rt250V	0	1	0	0	0	0
rt181T + rt250L	0	1	0	0	0	0
rt181V + rt250L + rt250V	0	0	0	1	0	0
rt204V + rt250L	0	1	0	0	0	0
rt207I	0	1	0	0	0	0

**Figure 5:**
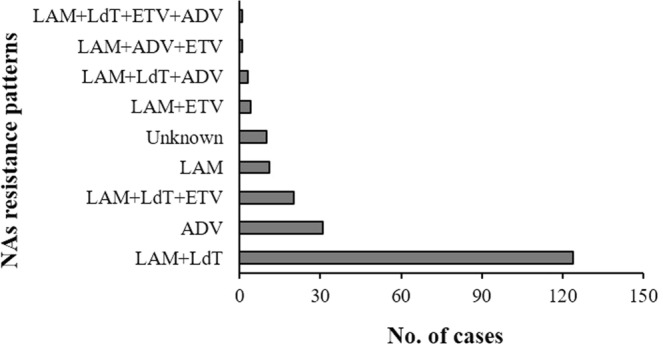
Patterns of NAs resistance happened in HBV patients in Shenzhen, China, 2015–18

NAs resistance analysis was carried out to evaluate five widely used NAs in Shenzhen—namely LAM, LdT, ADV, ETV, and TDF—according to EASL 2017 Clinical Practice Guidelines on the management of hepatitis B virus infection^21^. The overall NAs resistant occurrence rate was high, up to 17.03% (205/1204) (Table 4). There were 8 NAs resistance patterns, including 2 single NA resistance patterns and 6 cross NAs resistance patterns. It is noteworthy that there were several cases with mutation patterns that were not included in the guideline. NAs resistance patterns included the following: LAM + LdT cross resistance, accounting for 60.49% (124/205); ADV single resistance pattern, accounting for 15.12% (31/205); LAM + LdT + ETV cross resistance occurrences, accounting for 9.76% (20/205); other NAs resistance patterns were rare (Fig. 5).

**Table 4:** Distribution of NAs resistance patterns in different HBV genotypes in Shenzhen, China, 2015–18.

Genotypes/NAs resistance patterns	B	C	D	mixed B + C	mixed B + D	Unclassified
LAM + LdT	86	35	0	1	1	1
ADV	21	8	0	1	0	1
LAM + LdT + ETV	14	6	0	0	0	0
LAM	4	7	0	0	0	0
Unknown	5	5	0	0	0	0
LAM + ETV	3	1	0	0	0	0
LAM + LdT + ADV	1	2	0	0	0	0
LAM + ADV + ETV	1	0	0	0	0	0
LAM + LdT + ETV + ADV	1	0	0	0	0	0

## Discussion

HBV infection in China is an enormous economic and social burden^21^. Global prevalence of HBV infection varies considerably and is divided into high (>8%), intermediate (2–7%), and low (<2%) endemic areas. Although China has adopted a comprehensive strategy to control HBV and has successfully lowered the prevalence of HBV over the past 3 decades, it still remains a serious health threat in many regions that have high HBsAg prevalence^22–25^. Using the largest sample size to date (n = 282,166), we investigated the prevalence of HBV in Shenzhen from Aug. 26, 2015 to Sept. 11, 2018. Not surprisingly, Shenzhen was also a high endemic area with a HBsAg prevalence of 9.69%. Moreover, the HBV-DNA positive rate reached a peak of 57.97% in the 34,368 cases involved in serum HBV-DNA detection. Consistent with reports from other areas, the most infectious age was mainly distributed in the above 20 years age groups, accounting for 98.66%, with males recorded a greater proportion^26–28^. However, this investigation cannot explain the causes for this high prevalence of HBV in this area, since the means of infection in this large-scale patient study are unknown and could not be analyzed. That there are only 1.35% (369/27336) HBsAg positive cases aged under 20 years demonstrates that the vaccination plan in the last two decades significantly reduced HBV infection. Older patients, who were more likely not to be vaccinated and had high-risk behaviors (including sexual behavior and taking drugs that could cause HBV spread though mouth mucosa, genital mucosa or blood) could be the main cause of this high HBV rate.

It is worth explaining that HBV infection varied greatly with age, which showed 6.42% HBV prevalence in the younger than 1 age group. This data for younger than 20 age groups could just reflect the fact that Shenzhen People’s Hospital is an adult hospital and receives few children or teenager patients, who more often would go to a children’s hospital, and thus the sample size was extremely small.

Historically, newborns are required to be injected with the HBV vaccine and the vaccine-induced immunity, shown by HBsAb, was spontaneously highest at 79.72% in the infants’ group. However, nearly 40% of teenagers, 35% of children, and 30% of adults, had no immune protection. These results strongly support that persons without HBV infection, in all age groups, should obtain vaccine or booster shots.

Since HBV genotype detection is not required in the current guidelines of HBV management^29^, only 6.04% (1204/19922) of the infected population with HBV DNA positive had the genotype identified. However, many studies have demonstrated that different genotypes have different clinical features and outcomes, and therefore detection is extremely important in the evaluation of prognosis and treatment^18,30,31^. In addition, as infectious agents, HBV genotype distribution has geographical features^15–17,32,33^. Based on the large-scale analysis in this study, genotype B was found predominant in Shenzhen, and genotype C was also common, whereas genotype D was rare. B/C and B/D recombinant infections were also present, even though the frequencies were very low. It is noteworthy that a few HBV infection cases were found to be infected with other genotypes, which suggested that better classification methods, which can detect more genotypes (such as sequencing) are needed in the Shenzhen clinical laboratory.

NAs-treated HBV patients have a risk of developing drug resistant mutations, the occurrence of which, while often reported worldwide, varied greatly in areas^16,34–36^. NAs-resistance could lead to treatment failure and even cross-resistance^37^. The knowledge of resistance is indispensable for the selection of initial therapy or to a subsequent remedial treatment. To investigate drug resistance in Shenzhen, we further investigated all of the 1204 HBV patients who had been tested in the Shenzhen People’s Hospital during the past three years.

The results showed that the HBV mutation rtM204I/V together with rtL180M were the three mutations with the highest incidence, thus demonstrating that LAM-resistance and LdT were the most common resistances in Shenzhen. However, it is noteworthy that the mutation rt181V was considered to be a cross-resistant mutation, which could also induce LAM-resistance and LdT-resistance. NAs resistant mutation occurrence patterns were multitudinous in this study, including single mutation patterns and combinational mutation patterns. Single mutation patterns accounted for the majority. Combinational mutation occurrences were mainly L180M + M204I/V. Therefore, NAs mutations should be detected and carefully analyzed for patients.

The overall NAs resistance rate was still high and patterns were multiple. Cross resistance patterns were in the majority, in which LAM + LdT and LAM + LdT + ETV cross resistance occurrences accounted for 70.24% of all 205 NAs resistant cases. Single resistance pattern mostly occurred in ADV, accounting for 15.12%. Resistance to ETV, a potent antiviral drug with a higher genetic barrier, is considered to be rare, but in fact, it had an incidence of 12.20% (25/205) in combinational NAs resistance patterns. Therefore, when cross-mutations are observed, clinicians should carefully evaluate the possibility of NAs resistance and prescribe the proper antiviral drug to achieve a better outcome.

In addition, surprisingly, it was observed that drug resistance-associated HBV mutations occurred markedly more frequently in males, accounting for 80.7% of the total cases. As shown in our study and other reports, there is no significant gender difference in HBV infection^38^. Our study cannot explain the exact reasons for the notable gender difference in the incidence of drug resistant mutations. However, this might occur because males had lower self-discipline or habits that might cause them to neglect proper and timely medication, which could increase drug resistance.

Altogether, our results demonstrate that the epidemic situation of HBV is still critical in Shenzhen, as is NAs-resistance. Lacking HBV genotype and mutation information, we strongly advocate simultaneous detection of them in the clinical laboratory center, as well as the related serological factors of antigens, antibodies, and DNAs. This procedure will greatly enhance the understanding of the molecular epidemiology of HBV and provide more evidence for clinical determination. We also recommend that male patients follow medical advice and pay special attention to daily medication.
